# Amoxicillin-metronidazole disk for antimicrobial susceptibility testing of subgingival bacteria

**DOI:** 10.1080/20002297.2025.2508479

**Published:** 2025-05-23

**Authors:** Anne Birkeholm Jensen, Ellen Frandsen Lau, Niels Nørskov-Lauritsen

**Affiliations:** a Department of Dentistry and Oral Health, Aarhus University, Aarhus, Denmark; b Department of Clinical Microbiology, Esbjerg Hospital University Hospital of Southern Denmark, Esbjerg, Denmark

**Keywords:** Periodontitis, antimicrobial resistance, disk diffusion method, periodontal pathogens, oral microbiology

## Abstract

**Background:**

Antibiotics are used in periodontal therapy in selected cases, but therapy is rarely guided by antimicrobial susceptibility testing (AST). Direct AST of the oral microbiota using a combination disk with different antibiotics could provide a new way of AST to guide treatment planning.

**Methods:**

We performed AST of 46 strains of *Aggregatibacter actinomycetemcomitans*, *Fusobacterium nucleatum complex*, *Prevotella species*, and *Porphyromonas gingivalis*, with a combination disk of amoxicillin (AMX) and metronidazole (MET). The AMX-MET was compared to the largest inhibition zone diameter (IZD) obtained with AMX or MET disks, using an ordinary least square linear regression model.

**Results:**

The IZD of the AMX-MET correlated with the AMX for *A. actinomycetemcomitans* (interception 0.3) and with the MET for *Fusobacterium* (interceptions −1.25). For *Prevotella*, the AMX-MET was compared to AMX and MET after 20 and 44 h resulting in a superior correlation after 20 h (interception 0.06 vs 6.61 after 44 h). For *P. gingivalis*, the AMX-MET was compared to MET after 44 h resulting in an inferior correlation (interception 16.65).

**Conclusion:**

The IZD of AMX-MET was comparable to that of AMX and MET for important periodontal pathogens, which opens for studies on direct AST of oral samples with a mixed microbiota.

**Key message:**

The amoxicillin-metronidazole disk for antimicrobial susceptibility testing results in comparable inhibition zone diameters to that of AMX and MET for important periodontal pathogens.

## Introduction

Most cases of periodontal disease involve a multi-microbial cause by interacting microbial species living in a biofilm on the root surface of the tooth [1]. The standard treatment of periodontitis is mechanical debridement to remove and disrupt the dysbiotic biofilm, in some cases supplemented with periodontal surgery [2]. A minor proportion of periodontal disease cases, however, continue to progress, and in those cases, antibiotic therapy may be considered to supplement standard therapy. The subgingival plaque of these cases has repeatedly been demonstrated to consist of numerous potentially pathogenic microorganisms, which makes antimicrobial susceptibility testing (AST) challenging. Consequently, narrow-spectrum antibiotics must be combined to address a broader spectrum of microorganisms, and the combination of metronidazole (MET) and amoxicillin (AMX) is the preferred supplementary antibiotic therapy [3].

AMX is a beta-lactam antibiotic with a bactericidal effect inhibiting the cell wall peptide-glycan cross-linking of most Gram-positive and many Gram-negative bacteria. MET is a prodrug that primarily destroys bacterial DNA in strict anaerobic bacteria (and protozoa) after intracellular activation by nitro reductases [4]. Thus, the two agents target different molecules, and the bacterial spectra are only partly overlapping. Resistance towards AMX is common in several oral and extra-oral species and may be caused by different mechanisms [5]. MET resistance is still relatively infrequent [4]. Often, resistance mechanisms towards MET comprise changes in the target site or lack of drug activation intracellularly [4].

Antimicrobial resistance in oral species necessitates a continued focus on how to improve the antimicrobial susceptibility testing of oral samples [6,7]. Microbiological analysis and AST prior to treatment is challenged by the polymicrobial nature of biofilms associated with periodontitis. The approach of performing AST directly on polymicrobial clinical samples of other origins has been described, often referred to as rapid or direct AST [8–10]. Direct testing of polymicrobial samples in a single assay using a combination disk with the frequently used AMX and MET may potentially identify resistant strains that must be subjected to individual AST.

In 2021, a method for AST of rapidly growing anaerobes following the guidelines by the European Committee on Antimicrobial Susceptibility Testing (EUCAST) was described [11], and EUCAST criteria and guidelines are freely available [12,13]. We tested this method on oral, anaerobic Gram-negative rods and found that modifications were needed for the assessment of moderately growing anaerobes [14].

The polymicrobial nature of subgingival plaque samples support the use of two antibiotics to address a broader spectrum of microorganisms. Before testing a combination disk with AMX and MET on a polybacterial sample, it is necessary to secure that the combination disk gives results compatible with a reference disk of the single antibiotic for each of the pathogenic subgingival bacteria. In the present study, we aimed to test an amoxicillin-metronidazole disk (AMX-MET disk) for AST of well-known periodontal pathogens.

## Materials and methods

### Bacterial strains and culture conditions

*Clostridium perfringens* (ATCC 13,124^T^) and *Bacteroides fragilis* (ATCC 25,285^T^ and BF018 (multi-resistant)) served as reference and quality control strains for the method. *A. actinomycetemcomitans* (ATCC 43,718) served as an internal control strain.

Nine *A. actinomycetemcomitans* strains, six *Fusobacterium nucleatum complex* strains, 20 strains of *Prevotella species*, and 11 *Porphyromonas gingivalis* strains, all from the culture collection at the Department of Dentistry and Oral Health, Aarhus University, DK, were included in the study (Table 1).

**Table 1.** Study strain collection.

Organism	Strain designation
*Aggregatibacter actinomycetemcomitans*	ATCC 33,384^T^
*Aggregatibacter actinomycetemcomitans*	CCUG 1210
*Aggregatibacter actinomycetemcomitans*	UK7
*Aggregatibacter actinomycetemcomitans*	UK11
*Aggregatibacter actinomycetemcomitans*	UK21
*Aggregatibacter actinomycetemcomitans*	UK42
*Aggregatibacter actinomycetemcomitans*	UK44
*Aggregatibacter actinomycetemcomitans*	PN603
*Aggregatibacter actinomycetemcomitans*	PN604
*Fusobacterium nucleatum*	ATCC 25,586^T^
*Fusobacterium nucleatum*	IOOS 135
*Fusobacterium periodonticum*	ATCC 33,693^T^
*Fusobacterium polymorphum*	ATCC 10,953^T^
*Fusobacterium polymorphum*	IOOS 98
*Fusobacterium pseudoperiodonticum*	IOOS 12
*Prevotella melaninogenica*	ATCC 25,845^T^
*Prevotella melaninogenica*	HG73
*Prevotella melaninogenica*	HG118
*Prevotella intermedia*	CCUG 24,041^T^
*Prevotella intermedia*	AH 8291-E
*Prevotella intermedia*	AHN 10,754
*Prevotella intermedia*	AHN 8290
*Prevotella intermedia*	AHN 8764
*Prevotella intermedia*	OMZ248
*Prevotella intermedia*	OMZ324
*Prevotella nigrescens*	CCUG 9560^T^
*Prevotella nigrescens*	AHN 8272
*Prevotella nigrescens*	AHN 8292
*Prevotella nigrescens*	OMZ251
*Prevotella nigrescens*	OMZ265
*Prevotella pallens*	NCTC 13,042^T^
*Prevotella pallens*	AHN 8275
*Prevotella pallens*	AHN 8404
*Prevotella pallens*	AHN 8431
*Prevotella pallens*	AHN 8858
*Porhyromonas gingivalis*	ATCC 33,277^T^
*Porhyromonas gingivalis*	ATCC 53,978
*Porhyromonas gingivalis*	EF14449
*Porhyromonas gingivalis*	HG184
*Porhyromonas gingivalis*	OMGS 946
*Porhyromonas gingivalis*	AHN 9176
*Porhyromonas gingivalis*	BH 6
*Porhyromonas gingivalis*	OMZ 409
*Porhyromonas gingivalis*	IOOS 577
*Porhyromonas gingivalis*	GH6
*Porhyromonas gingivalis*	PGF7 (W83)

The strains were subcultured anaerobically (80% N_2_, 10% CO_2_, 10% H_2_) on M6 in-house medium without antibiotics (a Brucella blood-based medium) [15] twice before being used for AST. The strains were used for AST after 48 h of incubation.

### Antibiotic disks

Amoxicillin disks (AMX; 2 µg) and metronidazole disks (MET; 5 µg) were obtained from Oxoid/Thermo Fischer Scientific, Basingstoke, UK. The combination disk (AMX-MET disk) was prepared by applying a MET solution to the standard AMX disks. From a stock solution (1000 mg/L) of MET (Sigma-Aldrich Chemie GmbH, Steinheim, Germany) in dimethyl sulfoxide (Sigma-Aldrich Chemie GmbH, Steinheim, Germany), 5 µl (corresponding to 5 µg) MET stock solution was added to a 2-µg AMX disk, thereby creating the AMX-MET (2:5 µg) disk. The largest inhibition zone diameter (IZD) obtained with either a MET disk or an AMX disk for individual strains was used for comparison with the AMX-MET disk. Inhibition induced by the weakest antimicrobial would not be visible due to the action of the stronger one unless the combination of the two antimicrobials expressed synergistic action.

### Antimicrobial susceptibility testing

The disk diffusion method for the anaerobic bacteria was carried out as previously reported with minor modifications [13,14]. IZDs of *Fusobacterium nucleatum complex* and *A. actinomycetemcomitans* were read after 20 h, *Prevotella* species after 20 and 44 h, and readings of *P. gingivalis* were restricted to 44 h. *A. actinomycetemcomitans* was tested according to EUCAST guidelines for *H. influenzae* (inoculum of 0.5 McFarland), except that FAA plates (Statens Serum Institute, SSI, Copenhagen, DK) were inoculated and the strains were incubated anaerobically due to the testing of metronidazole susceptibility, rather than Mueller–Hinton agar with horse blood and NAD (MH-F) and incubation in ambient air supplemented with 5% CO_2_. For anaerobic bacteria, FAA plates were inoculated with McFarland 1.0 and incubated anaerobically. IZDs were interpretated according to EUCAST criteria using the unaided eye [11,12]. AST was performed in triplicates.

### Data analysis

All data analyses were performed using the SciPy program (https://docs.scipy.org/doc/scipy/index.html). The analyses were made based on the results from the triplicates runs. However, for some strains, one out of three runs resulted in no growth after 20-h incubation. In that case, the analysis was made based on the mean of two runs.

The correlation between the IZDs of AMX-MET disk and reference disks was analysed using (an) ordinary least square linear regression model (OLS). The r^2^ describes the relationship between the two disks. However, the r^2^ is not sufficient to describe the correlation of two methods because it does not take bias into account. Therefore, the relationship between the two methods were primarily described by the slope and the interception of the OLS [16]. It indicates no bias in the model prediction, i.e. the relationship between the disks, if the plotted data do not scatter around the identity line. An intercept of 0.0 indicates that the regression model does not possess bias due to consistent errors. A slope >0.00 illustrates a proportional linear relationship between the two methods.

## Results

IZDs of control strains were within the expected range in all runs.

**Figure 1.**
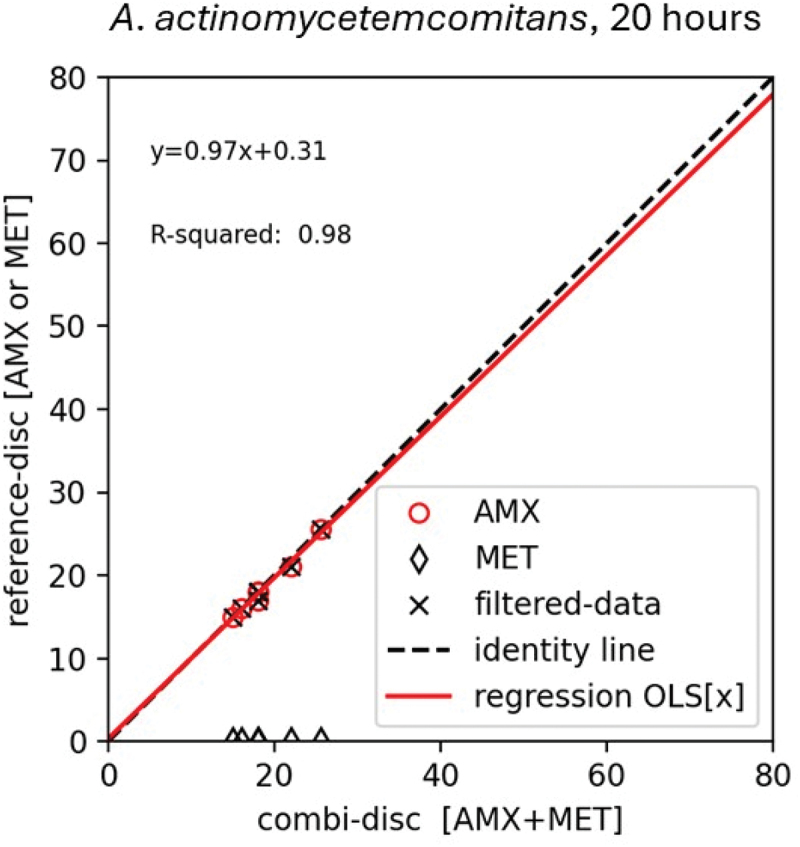
The relationship between the inhibition zone diameter in mm of the AMX-MET disk (x-axis) and reference disks (y-axis) determined by an ordinary least square linear regression model for nine *Aggregatibacter actinomycetemcomitans*. The relationship is illustrated by the regression line (red). The inhibitions zone obtained on the amoxicillin disk is illustrated by a red circle, on the metronidazole disk as a black rubric, and the AMX-MET disk as (filtered data) a black cross. The AMX disk resulted in the biggest inhibition zone diameters and served as reference disk for all *A. actinomycetemcomitans*.

There were no measurable inhibition zones around the MET disk for any of the *A. actinomycetemcomitans* strains, and the AMX disk therefore served as comparative disk. The AMX-MET disk showed good correlation to the AMX disk with an r^2^ of 0.98 after 20 h (Figure 1). The data did not scatter around the identity line, which in combination with an interception of 0.31 indicates no consistent bias in the relationship between the AMX-MET disk and the AMX disk. The slope of 0.97 indicates a good linear relationship.

**Figure 2.**
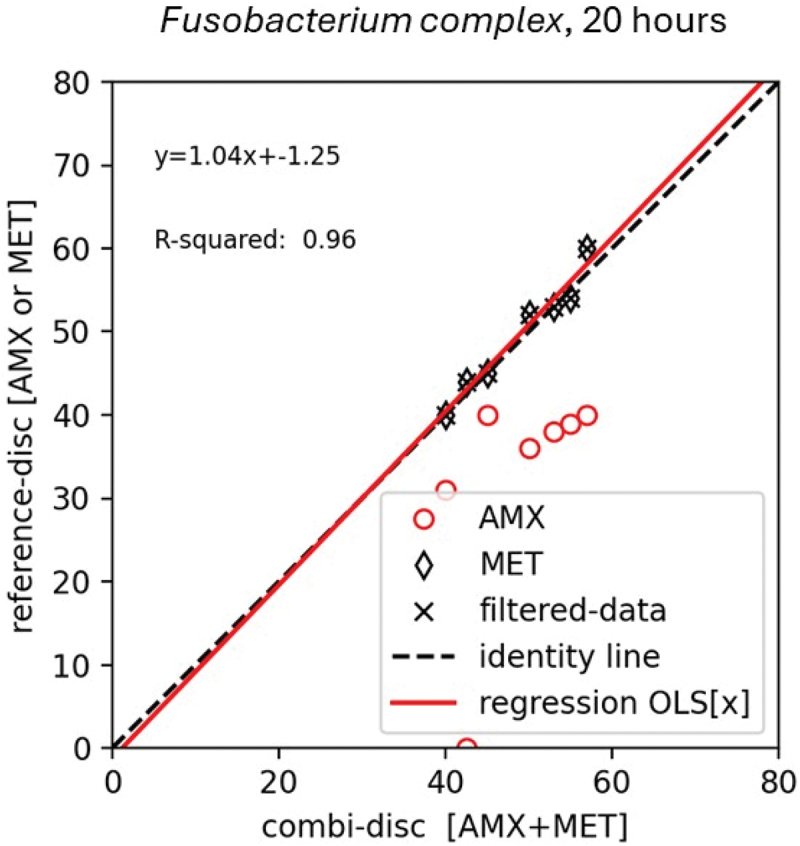
The relationship between the inhibition zone diameter in mm of the AMX-MET disk (x-axis) and reference disks (y-axis) determined by an ordinary least square linear regression model for six *Fusobacterium complex*. The relationship is illustrated by the regression line (red). The inhibitions zone obtained on the amoxicillin disk is illustrated by a red circle, on the metronidazole disk as a black rubric, and the AMX-MET disk as (filtered data) a black cross. The MET disk resulted in the biggest inhibition zone diameters and served as reference disk for all *Fusobacterium*.

The MET disk resulted in the largest IZDs for all the tested *Fusobacterium nucleatum complex* strains. The AMX-MET disk showed good correlation to the MET disk with an r^2^ of 0.96 after 20 h (Figure 2). The data did not scatter around the identity line, and the interception was −1.25 indicating no consistent bias in the relationship between the AMX-MET disk and the MET disk. The slope of 1.04 indicates a good linear relationship.

**Figure 3.**
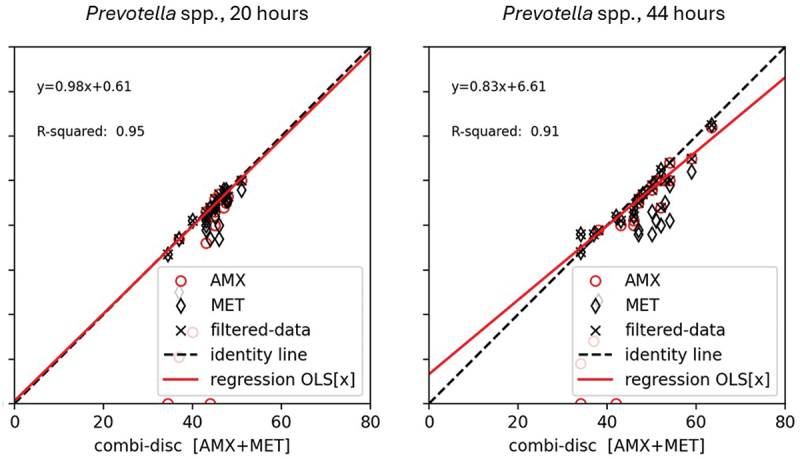
The relationship between the inhibition zone diameter in mm of the AMX-MET disk (x-axis) and reference disks (y-axis) determined by an ordinary least square linear regression model for 18 *Prevotella* spp. The relationship is illustrated by the regression line (red). The inhibitions zone obtained on the amoxicillin disk is illustrated by a red circle, on the metronidazole disk as ablack rubric, and the AMX-MET disk as (filtered data) a black cross. The AMX disk resulted in the largest IZD for seven strains and the MET disk for eleven strains, and the disks served as reference strains for the respected *Prevotella* strains.

Twenty *Prevotella* strains were tested in triplicate, but only 48 of 60 experiments were readable after 20 h. For 18 strains, more than one read was possible, and comparisons were made based on the mean of two or three runs. The AMX disk resulted in the largest IZD for seven strains and the MET disk for 11 strains. After 44 h, all 60 experiments were readable. The AMX disk had the largest IZD for twelve strains, and the MET disk for eight strains; the MET and AMX IZDs were similar for some of the strains, and the superior IZDs of AMX or MET for individual strains after 20 h could switch after prolonged incubation. Overall, the AMX-MET disk resulted in good correlation with an r^2^ of 0.95 and 0.91 after 20 h and 44 h, respectively (Figure 3). Figure 3 shows that the relationship between the AMX-MET disk and the reference disks after 20 h was better than after 44 h based on all parameters. After 20 h, the relationship between the disks did not contain consistent bias based on the interception of 0.61 and the data did not scatter around the identity line. The interception of 6.61 after 44 h indicates consistent errors in the relationship. Both relationships were linear.

**Figure 4.**
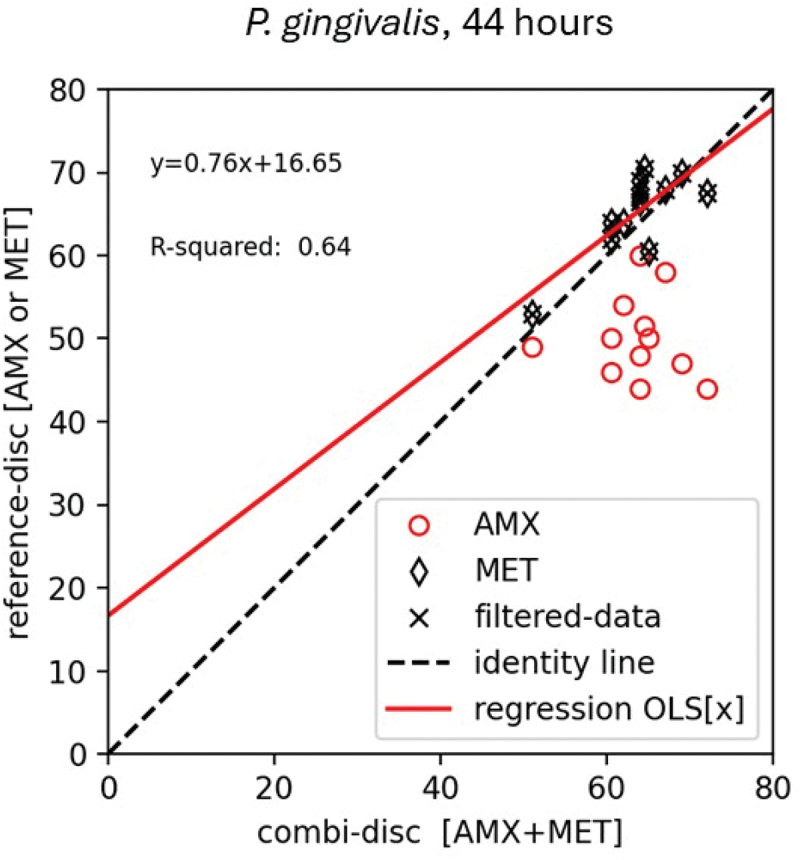
The relationship between the inhibition zone diameter in mm of the AMX-MET disk (x-axis) and reference disks (y-axis) determined by an ordinary least square linear regression model for eleven *Porphyromonas gingivalis*. The relationship is illustrated by the regression line (red). The inhibitions zone obtained on the amoxicillin disk is illustrated by a red circle, on the metronidazole disk as a black rubric, and the AMX-MET disk as (filtered data) a black cross. The MET disk resulted in the biggest inhibition zone diameters and served as reference disk for all *P. gingivalis*.

The MET disk resulted in the largest IZDs for all *P. gingivalis* strains. The r^2^ of 0.64 after 44 h indicates a reasonable relationship between the MET and the AMX-MET disks, however, the intercept of 16.65 indicates consistent errors (Figure 4). In addition, the data scattered around the identity line to a higher degree for *P. gingivalis*. Of the investigated species, *P. gingivalis* showed the poorest correlation between AMX-MET and the comparative disk.

**Figure 5.**
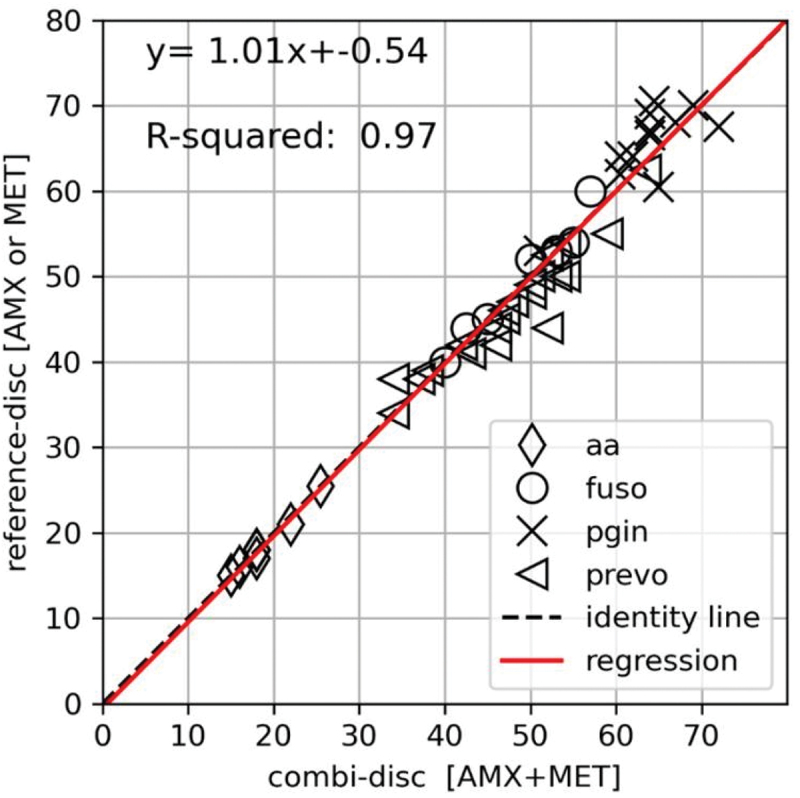
The relationship between the inhibition zone diameter in mm of the combination AMX-MET disk (x-axis) and reference disks (y-axis) determined by an ordinary least square linear regression model for 46 study strains including *Aggregatibacter actinomycetemcomitans* (aa, rubric), *Fusobacterium complex* (fuso, circle), *Prevotella* spp. (prevo, triangle), and *Porphyromonas gingivalis* (pgin, cross). The relationship is illustrated by the regression line (red).

Figure 5 shows the performance of the AMX-MET disk for all strains combined of the species tested in the study. The inhibitions zone diameters obtained with AMX-MET disk correlated to the reference disks with a slope of 1.01, an intercept of −0.54, and an r^2^ of 0.97 indicating an overall good correlation between the AMX-MET disk and the reference disks.

## Discussion

In the present study, we compared the IZD of an AMX-MET disk to the IZDs of an AMX or a MET disk. In a recently published systematic review and meta-analysis, the AMX-MET combination was the only antimicrobial treatment that was more effective than scaling and root planning in improving all clinical outcomes when used to treat periodontitis in combination with mechanical debridement or surgery [3]. To facilitate rapid AST of subgingival bacterial samples, we combined the two preferred antimicrobial agents for treatment of periodontitis in a single antimicrobial disk.

Our results showed a good correlation between the AMX-MET disk and the AMX disk for *A. actinomycetemcomitans* and between the AMX-MET disk and the MET disk for species of the *Fusobacterium nucleatum* complex. The EUCAST disk diffusion is not validated for *A. actinomycetemcomitans* and for *Fusobacterium* sp. other than *F. necrophorum*. However, the strains resulted in sufficient growth and readable IZD after 20 h on FAA, and the comparison of the AMX-MET disk to the individual antibiotic disks was possible. Among the *Prevotella* spp., the IZDs of the AMX-MET disk were compared to the AMX disk for some of the strains and to the MET disk for others, which may indicate variations in the susceptibility patterns towards AMX and MET within the *Prevotella* genus. For some of the *Prevotella* strains, the MET og AMX IZDs were similar, and the largest IZD could switch between the agents during prolonged incubation. According to EUCAST guidelines, *Prevotella* spp. tested with the method published by Bavelaar et al. should be interpreted after 20 h of incubation [17]. Prolonged incubation may lead to more variation in the IZD between reproduced runs. We decided to interpret results after 20 and 44 h because a considerable amount of the strains did not result in sufficient growth after 20 h, but the needs for prolonged incubation came at the expense of the correlation between the AMX-MET disk and the AMX or MET disk. The correlation was good after 20 h based on an interception of 0.61 but decreased after 44 h where more consistent errors were observed expressed by an interception of 6.61. However, even after 44 h incubation the correlation between the AMX-MET disk and reference disks was acceptable.

The disk diffusion method is not validated for *P. gingivalis*, and prolonged incubation is necessary because *P. gingivalis* has insufficient growth and unreadable IZD after 20 h incubation [14]. The correlation between the AMX-MET disk and the MET disk for *P. gingivalis* showed consistent errors expressed by an interception of 16.65 and scattering of the results around the identity line. The consistent errors may be explained by the disk diffusion method not applying ideally to *P. gingivalis*. However, the correlation between the AMX-MET disk and the MET was reasonable with an r^2^ of 0.61 and a linear relationship, and we believe that the AMX-MET disk may still be valuable for initial testing of *P. gingivalis* for selected purposes.

Direct AST of subgingival plaque samples on agar plates incorporated with breakpoint concentrations of doxycycline has shown a good correlation to the percentage of bacteria inhibited by standard MIC-producing procedures [18]. However, the quality of direct AST is debatable due to, e.g. the risk of an inoculum effect, difficulty in identification of species, and lack of standardization of the sample concentration [10]. The agar dilution method would be the preferred method for direct AST of dental samples because the EUCAST disk diffusion method has not been described for many oral microorganisms, but the dilution tests are expensive and time-consuming. On the other hand, the disk diffusion method is quick, reproducible and sustainable, and merits consideration for polymicrobial samples [19]. The AMX-MET disk indeed showed a very good correlation to the reference disk for all strains, and direct AST using a combined AMX-MET disk could be a rapid method to screen polymicrobial samples for resistant strains. AMX and MET target different microorganisms, and multiple resistant strains will likely occur in the inhibition zone if single disks are used for direct AST of a polymicrobial sample. The combination of two antibiotics in a single disk provides the opportunity to detect isolates that are not inhibited by any of the preferred drugs. Strains cannot be categorized as resistant or susceptible by direct AST with the combination disk, but relevant species can be selected and further tested following AST criteria, e.g. EUCAST or the Clinical & Laboratory Standards Institute (CLSI) criteria and guidelines.

Direct AST on the polymicrobial subgingival biofilm does not take into account the many as yet uncultivable microorganisms. At present, to include these organisms may require combining the direct AST with molecular-based methods detecting antimicrobial resistance genes (ARGs) [20,21]. Detection of ARGs may be insufficient for treatment planning because genotype and phenotype not necessarily correlate [22]. However, combining the different approaches for AST of the polymicrobial biofilm would result in a more comprehensive understanding of the biofilms antimicrobial nature [23].

The combination of AMX and MET has been reported to have a synergistic effect *in vivo* and *in vitro* on *A. actinomycetemcomitans* and *A. actinomycetemcomitans*-associated periodontitis [24,25]. The previously reported synergistic effect has been claimed to be caused by an enhanced cellular uptake of metronidazole and its hydroxy metabolite in the prescence of AMX [26]. If the AMX-MET disk resulted in a significant larger zone than the reference disk, this could indicate a synergistic action of the combination of amoxicillin and metronidazole. The AMX-MET disk did not result in larger IZDs than the reference disk indicating no in vitro synergistic effect of the combination of AMX and MET against the nine tested *A. actinomycetemcomitans* isolates.

The lack of MIC determination of the strains might be considered as a limiting factor. However, the aim was to compare the inhibition zone diameter of the different disks. Future studies testing the AMX-MET disk on polymicrobial samples should include MIC determination of the strain collection that should in addition include resistant strains.

In conclusion, our study has demonstrated that the AMX-MET disk has inhibition zone diameters comparable to AMX or MET disks for several oral species. The AMX-MET disk could potentially be used for direct AST to analyse subgingival plaque samples from periodontitis patients.
